# A Coexisting Pilocytic Astrocytoma and a Prolactinoma: A Case Report of Collision Tumors and Literature Review

**DOI:** 10.7759/cureus.4911

**Published:** 2019-06-17

**Authors:** Antonia Malli, Savvas Melissaris, Anastasia Dimitriadi, Theodosia Choreftaki, Nikolaos Georgakoulias

**Affiliations:** 1 Department of Neurosurgery, The National and Kapodistrian University of Athens School of Health Sciences, Athens, GRC; 2 Department of Neurosurgery, General Hospital of Athens "G. Gennimatas", Athens, GRC; 3 Department of Pathology, General Hospital of Athens "G. Gennimatas", Athens, GRC

**Keywords:** collision tumors, prolactinoma, pilocytic astrocytoma

## Abstract

Pituitary adenomas and gliomas constitute two of the most common primary intracranial tumors. However, their coexistence as collision tumors is relatively rare and few similar reports could be identified in the literature. In this study, we report a case of a 64-year-old male patient with a prolactinoma and a pilocytic astrocytoma in collision. The patient underwent both an endoscopic transsphenoidal approach and a subfrontal craniotomy, achieving a gross total resection of the concomitant lesions in the sellar and suprasellar regions. Postoperatively, the patient's preoperative bitemporal hemianopsia resolved and no new deficits occurred. At his six-month follow-up, he remained free of neurologic deficits. Although causative factors are yet to be determined for these tumors in collision, their nonsyndromic coexistence could point to a common genetic linkage which will help to shed light on their natural history of occurrence.

## Introduction

Pilocytic astrocytomas account for 5.4% of all gliomas, with an incidence much higher in the pediatric population [1]. In general, pilocytic astrocytomas typically correspond to the World Health Organization (WHO) Grade I tumour, arising both in the supratentorial and infratentorial regions [1]. Pituitary adenomas are one of the most common lesions in the sellar area, and they are generally classified into functioning and nonfunctioning neoplasms. Lactotrophic tumors (prolactinomas) constitute the most prevalent secreting pituitary adenomas [2-3]. The coexistence of distinct primary neoplastic entities in the same intracranial region is generally defined as tumors in collision [4-5]. The collision of pituitary adenomas and gliomas has been previously reported in the existing literature [6-9]. However, to our best of knowledge, this is the first study to identify the coexistence of a prolactinoma and a pilocytic astrocytoma and their potential common pathogenetic mechanism.

## Case presentation

**Figure 1:**
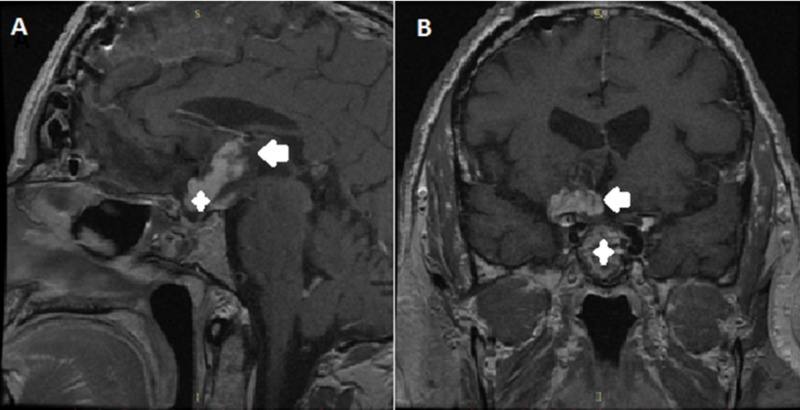
Preoperative magnetic resonance imaging (MRI) of the brain (A) Sagittal contrast-enhanced T1-weighted image showing a heterogeneously enhancing mass in the sellar (white cross), suprasellar, and hypothalamic (white arrow) regions; (B) coronal contrast-enhanced T1-weighted scan demonstrating two different heterogeneous lesions with cystic components in the sellar (white cross), suprasellar, and hypothalamic (white arrow) regions.

**Figure 2:**
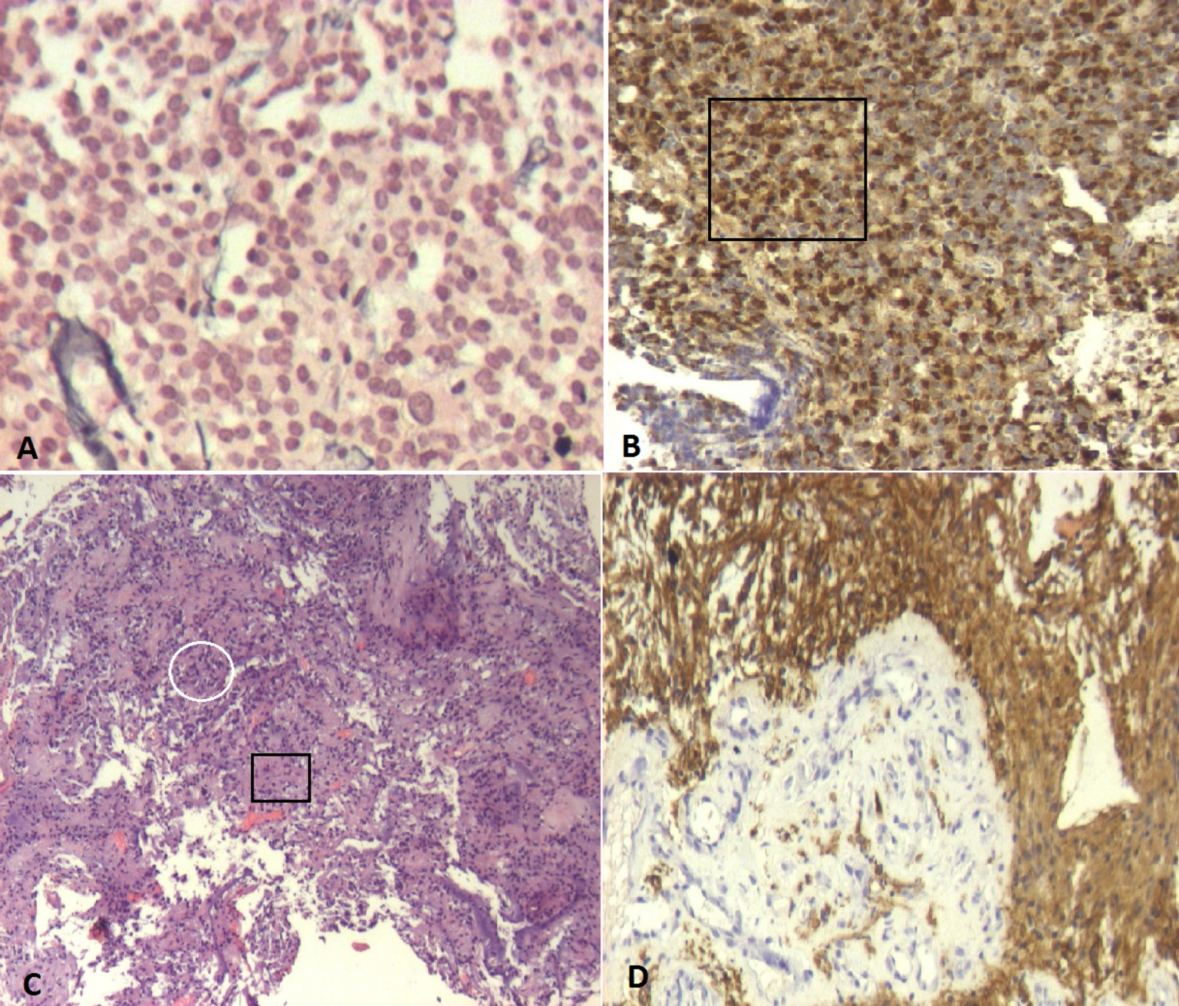
Histopathological examination after surgical resection (A) Histochemistry shows a pituitary adenoma, reticulin staining, which shows a distortion in the reticulin network (x100); (B) immunohistochemistry shows positive staining for prolactin (black frame, x100); (C) hematoxylin-eosin staining reveals an astrocytic tumor of low (black frame) to moderate (white circle) cellularity with a biphasic pattern as seen in pilocytic astrocytomas (x50); (D) positive immunohistochemical staining for glial fibrillary acidic protein (GFAP, x100).

A 64-year-old male presented in our Neurosurgery Department with a 12-month history of visual disturbances, including bitemporal hemianopia. No history of prior radiation, chemical substance exposure, or associated familial tumor syndromes were reported. Magnetic resonance imaging (MRI) showed a suprasellar, not well-delineated tumor (36 x 29 x 25 mm in diameter) with both cystic and solid components, which expanded into the third ventricle and had a heterogeneous enhancement in its periphery (Figure 1). The tumor pressed against the optic chiasm and surrounded the A1 segment of the right anterior cerebral artery. There was also another lesion located in the left part of the adenohypophysis with a maximum diameter of 12 mm. Upon laboratory testing, serum prolactin levels were significantly increased above normal at 313 ng/dl (normal range up to 5 ng/dl), and thus, he was treated with cabergoline, which only led to a minimal clinical improvement of the patient. Surgical intervention was then considered as the best treatment approach. Subsequently, both an endoscopic transsphenoidal approach and a subfrontal craniotomy were performed, achieving gross total resection of the lesions located in the sellar, the suprasellar, and hypothalamic regions. Histology of the sellar lesion revealed a pituitary adenoma with positive immunohistochemistry for prolactin, whereas the suprasellar/hypothalamic part of the tumour was histologically identified as a pilocytic astrocytoma (Figure 2). Postoperatively, the patient had no visual loss and thus improved neurologically with no neurologic deficit. At six months follow-up, he was symptom-free.

## Discussion

Pituitary adenomas and astrocytomas in collision are a very rare occurrence reported in the current literature (Table 1). Our search in PubMed Central (PMC) and Google Scholar revealed no other case of a prolactinoma and a pilocytic astrocytoma coexisting in the same intracranial area. To our knowledge, this is the first study that describes such a combination of neoplasms.

**Table 1:** Literature Review for the Coexistence of Pituitary Adenomas and Astrocytomas *information on the type of surgery was not available

Study	Sex,Age	Tumor		Treatment
Naik et al. [6]	Male, 36 years old	Pituitary macroadenoma; anaplastic astrocytoma	Gross total resection via modified pterional craniotomy	
Jaiswal et al. [7]	Female, 48 years old	Ossifying pituitary adenoma; low-grade astrocytoma	Gross total resection via left frontotemporal craniotomy	
Furtado et al. [8]	Male, 35 years old	Thyrotropin-secreting pituitary adenoma; low-grade astrocytoma	Gross total resection via transsphenoidal endoscopic surgery and right frontal craniotomy	
Ezura et al. [9]	Male, 33 years old	Pituitary adenoma (chromophobe); anaplastic astrocytoma	Gross total resection*	

The pathogenesis of prolactinoma and pilocytic astrocytoma in collision still remains undefined. However, several hypotheses could be valid. (1) This co-existence as a single event could be a coincidence of two common brain neoplasms. (2) Abnormalities in the BRAF gene and its signaling mitogen-activated protein kinase (MAPK) pathway have been identified as the most prominent molecular changes occurring in pilocytic astrocytomas [1]. However, the N-myc downstream-regulated gene 2 (NDRG2), known as a tumor suppressor gene, could serve as a common pathogenetic factor for prolactinomas and pilocytic astrocytomas. In general, NDRG2 is located at chromosome 14q11.2. Skiriute et al. revealed that NDRG2 expression increased in glial cells in Grade I or II astrocytomas, while its expression was significantly decreased in higher grades [10]. Moreover, the latest evidence shows a promising genetic association between prolactinomas and the NDRG2 gene. The study by Vaitkiene et al. presented a higher expression of the NDRG2 gene in prolactinomas compared to all the other types of both functioning and non-functioning pituitary adenomas [11]. (3) Recent studies have shown that steroid hormones, especially estrogens, may play an essential role in the development and growth of human astrocytes [12-13]. It is well known that the majority of the estrogen effects are mediated by two types of nuclear receptors and transcriptional factors, ERα and ERβ. Although highly homologous, ERα and ERβ are encoded by separate genes located on chromosome 6q25.1 and 14q22-24, respectively [12]. Studies have shown that estradiol-mediated activation of ERα, as well as the recruitment of steroid receptor coactivators (SRC-1 and SRC-3), led to astrocytoma growth [13-15]. Moreover, other studies have demonstrated increased expression of ERα in prolactinomas, a phenomenon which could possibly promote tumorigenesis by mediating autocrine or paracrine actions of the basic fibroblast growth factor (bFGF) and vascular endothelial growth factor (VEGF) [16-18].

Another key concept seen in vitro and in vivo is the expression of the pituitary tumor transforming gene (PTTG) induced by estrogens and bFGF, enabling the proliferation of lactotrophs and tumor formation [16, 18]. The presence of increased ERα in both prolactinomas and pilocytic astrocytomas could be attributed to a common pathogenetic substrate that will help shed light on the pathophysiology of two seemingly unrelated tumors. This mechanism will also highlight new therapeutic strategies, such as estrogen receptor antagonists as part of the nonoperative management. Thus, primary studies need to be conducted in an experimental and clinical setting in order to assess the validity of the hypothesis of a common genetic linkage.

## Conclusions

Collision tumors consisting of a prolactinoma and a pilocytic astrocytoma are a rare phenomenon. While no causative factors can be derived from a single case report, we propose that potential mechanisms of this phenomenon are both ERα-induced and NDRG2-mediated proliferation of both astrocytes and lactotrophs.
